# DAILY CONSUMPTION OF KETONE ESTER, BIS-OCTANOYL (R)-1,3-BUTANEDIOL, IS SAFE AND TOLERABLE IN HEALTHY OLDER ADULTS

**DOI:** 10.1093/geroni/igae098.3646

**Published:** 2024-12-31

**Authors:** Chatura Senadheera, Traci Blonquist, Brianna Stubbs, Stephanie Roa Diaz, Sawyer Peralta, Elizabeth Stephens, John Newman

**Affiliations:** Buck Institute, Novato, California, United States; Biofortis Mérieux NutriSciences, Addison, Illinois, United States; Buck Institute, Novato, California, United States; Buck Institute, Novato, California, United States; Albert Einstein College of Medicine, New York, New York, United States; Buck Institute for Research on Aging, Novato, California, United States; Buck Institute for Research on Aging, Novato, California, United States

## Abstract

Ketone bodies are endogenous metabolites produced during fasting or a ketogenic diet that have pleiotropic effects on aging pathways. Ketone esters (KEs) are compounds that induce ketosis without dietary changes, but KEs have not been studied in an older adult population. The primary objective of this pilot study was to assess tolerability and safety of KE ingestion in older adults. We carried out a randomized, placebo-controlled, double-blinded, parallel-arm trial (NCT05585762) with community-dwelling, independent older adults in stable health. N=30 (M=15, F=15; age=76y, range 65 –90y) were randomized and n=23 completed. Participants were randomly allocated to consume either KE (25g bis-octanoyl (R)-1,3-butanediol) or a taste, appearance, and calorie-matched canola oil placebo (PLA) daily for 12 weeks. Tolerability was assessed using a daily log for 2-weeks, and then via a bi-weekly phone interview. Safety was assessed by vital signs and lab tests at screening and weeks 0, 4 and 12, along with tabulation of adverse events. There was no difference in the prespecified primary outcome of proportion of participants reporting moderate or severe nausea, headache, or dizziness on more than one day in a two-week reporting period (KE n=2 (14.3% [90% CI=2.6–38.5]);PLA n=1 (7.1% [90% CI=0.4–29.7]). Dropouts numbered four in the PLA group and two in the KE group. More symptoms were reported in both groups during the first two weeks; symptoms were reported less frequently between 2–12 weeks. There were no clinically relevant changes in safety labs or vital signs in either group.

